# Effects of High Dissolved Inorganic and Organic Carbon Availability on the Physiology of the Hard Coral *Acropora millepora* from the Great Barrier Reef

**DOI:** 10.1371/journal.pone.0149598

**Published:** 2016-03-09

**Authors:** Friedrich W. Meyer, Nikolas Vogel, Karen Diele, Andreas Kunzmann, Sven Uthicke, Christian Wild

**Affiliations:** 1 Department of Ecology, Leibniz Center for Tropical Marine Ecology (ZMT), Bremen, Germany; 2 Faculty of Biology and Chemistry, University of Bremen, Bremen, Germany; 3 Australian Institute of Marine Science, Townsville MC, Queensland, Australia; 4 School of Life, Sport and Social Sciences, Edinburgh Napier University, Edinburgh, United Kingdom; 5 St Abbs Marine Station, St Abbs, United Kingdom; National Taiwan Ocean University, TAIWAN

## Abstract

Coral reefs are facing major global and local threats due to climate change-induced increases in dissolved inorganic carbon (DIC) and because of land-derived increases in organic and inorganic nutrients. Recent research revealed that high availability of labile dissolved organic carbon (DOC) negatively affects scleractinian corals. Studies on the interplay of these factors, however, are lacking, but urgently needed to understand coral reef functioning under present and near future conditions. This experimental study investigated the individual and combined effects of ambient and high DIC (*p*CO_2_ 403 μatm/ pH_Total_ 8.2 and 996 μatm/pH_Total_ 7.8) and DOC (added as Glucose 0 and 294 μmol L^-1^, background DOC concentration of 83 μmol L^-1^) availability on the physiology (net and gross photosynthesis, respiration, dark and light calcification, and growth) of the scleractinian coral *Acropora millepora* (Ehrenberg, 1834) from the Great Barrier Reef over a 16 day interval. High DIC availability did not affect photosynthesis, respiration and light calcification, but significantly reduced dark calcification and growth by 50 and 23%, respectively. High DOC availability reduced net and gross photosynthesis by 51% and 39%, respectively, but did not affect respiration. DOC addition did not influence calcification, but significantly increased growth by 42%. Combination of high DIC and high DOC availability did not affect photosynthesis, light calcification, respiration or growth, but significantly decreased dark calcification when compared to both controls and DIC treatments. On the ecosystem level, high DIC concentrations may lead to reduced accretion and growth of reefs dominated by *Acropora* that under elevated DOC concentrations will likely exhibit reduced primary production rates, ultimately leading to loss of hard substrate and reef erosion. It is therefore important to consider the potential impacts of elevated DOC and DIC simultaneously to assess real world scenarios, as multiple rather than single factors influence key physiological processes in coral reefs.

## Introduction

There is concern about the effects of human-induced increases in atmospheric CO_2_, which is resulting in increasing dissolved inorganic carbon concentration (DIC) in the world’s oceans. This causes ocean acidification (OA) [1]. The rate of increase of DIC seawater concentration is unprecedented for the last 300 million years [2–5] and will very likely rise further [6] as the partial pressure of CO_2_ (*p*CO_2_) is increasing in both atmosphere and the ocean. The resulting reduced pH changes the carbonate system of the seawater by decreasing the saturation state of the different calcium carbonate components [7]. This ultimately affects many coral reef calcifying invertebrates such as hard corals, mollusks, echinoderms and foraminifera [8–13] and may lead to changes in calcification, productivity and benthic community structure of coral reefs [14–18]. An increased DIC content can lead to reduced photosynthesis rates of corals [14], increased respiration rates, altered symbiosis and reduced calcification [19–22] even at the larval stage [23–26]. In summary, these negative effects can lead to future reef decalcification as shown by mesocosm [27] or field studies [17,28,29].

Recent findings suggest that future predictions of *p*CO_2_ levels in seawater are likely a conservative estimate for highly productive areas such as coral reefs in coastal zones, where large natural variability of the carbonate chemistry [30], coupled with a decrease in buffer capacity, can amplify predicted future *p*CO_2_ concentrations up to three fold [31].

While DIC availability directly affects coral reef calcifiers, increased dissolved organic carbon (DOC) availability indirectly influences corals by altering coral-associated microbial communities and stimulating microbial activity [32–35]. However, so far no studies focused on the physiological response of corals towards high DOC concentrations. Thus, scientific knowledge is lacking. Main sources of exogenous DOC are sewage waters [36] and terrestrially derived sediments carrying high amounts of particulate organic carbon that can be transformed into dissolved organic material via microbial degradation [37]. Inorganic nutrients support micro- and macro-algae growth which in turn leads to increased DOC production (up to 1000 μmol L^-1^, [32]) during bloom and algae mat formation (up to 130 μmol L^-1^ [38]). For the Australian Great Barrier Reef (GBR), high inputs of inorganic nutrients and increased sediment loads through human activity have led to so called “phase shifts” and changed many coral-dominated to algae-dominated reef communities [39–41].

Nutrient and sediment inputs show high spatial and temporal variation. Land-derived inputs are particularly frequent during the wet season when precipitation is higher, and storm events are recurrent. Nutrient concentrations are particularly high in areas with high river discharges [37,42,43]. Both, agricultural land-use and area extent as well as strong storm events and floods likely increase further in the near future [44,45]. Therefore, the contribution of river discharge to DOC availability in the GBR will likely rise, as riverine runoff from agricultural influenced areas is one of the main sources of elevated DOC concentrations. In addition, inorganic nutrients may fuel benthic algal growth. Ensuing phase shifts may further increase bio-available DOC production [46,47] and its availability to microbial communities [46,48–53]. These compounds may also promote the growth of pathogens leading to coral bleaching and potential rapid coral mortality [32–34,54,55]. Finally, high microbial activity and degradation of organic carbon reduces the availability of oxygen for the coral holobiont to potentially critical levels [35,38,46,52].

Given the urgent need for understanding coral reef functioning and vulnerability in the Anthropocene, it is surprising that no studies on the combined effects of the important parameters DOC and DIC have been conducted to date. Studies on the effects on coral physiology, i.e. growth, calcification, and photosynthesis, are lacking, but crucial for evaluating the effects of non-lethal exposure to elevated DOC availabilities. In addition, high DOC availability may change the microbial communities associated with the coral holobiont and possibly reduce calcification and photosynthetic rates. In combination with high DIC availability, this may result in cumulative negative effects as high DIC may reduce calcification of corals [14,27].

The present study thus investigated the effects of combined high DIC and DOC exposure on a scleractinian coral in a laboratory experiment. We selected *Acropora millepora* (Ehrenberg, 1834), a common coral species from the GBR of which the response towards elevated DIC has been studied on the gene expression level [19,56], and effects of elevated DIC on early development and settlement have been described [23]. We monitored photosynthetic performance as well as growth throughout the experiment over 16 d and measured calcification, oxygen and nutrient fluxes as well as chl *a* (chlorophyll a) and protein content at the end of the experiment.

## Material and Methods

### Specimen collection and preparation

Colonies of the coral *Acropora millepora* (Ehrenberg, 1834) were collected from reefs next to Pelorus Island (S 18° 33.001’, E 146° 29.304’) in 2012 under a GBMPA sampling permit to the Australian Institute of Marine Science (AIMS). The colonies were fragmented using commercial pliers, and individual nubbins (3 to 4 cm height) glued onto ceramic stubs with superglue. Nubbins were mixed from different colonies and maintained in natural seawater flow-through aquaria (volume of several hundred liters) facilities at AIMS under plasma lights (150 μmol photons m^-2^ s^-1^) in a 12 h/ 12 h light-dark cycle for 3 months to adjust to laboratory conditions and allow to recover from fragmentation until using them for the experiment (see next section).

### Experimental setup

Two weeks prior to the onset of the manipulative experiment, 24 nubbins were randomly assigned into 12 experimental tanks (flow-through tanks with 18 l volume each). The experiment itself was conducted over a period of 16 d between 24 July and 9 August 2012 at AIMS. Three replicate tanks for the two treatments with two treatment levels were placed in alternating order. The treatments were *p*CO_2_ in ambient and high availability (403 μatm and 996 μatm, respectively) and DOC in ambient and high availability (83 ± 10 and 294 ± 506 μmol L^-1^ with DOC added as Glucose, D-Glucose, Sigma Aldrich, purity > 99,5% in 0 and 377 μmol L^-1^).

### DOC & DIC treatment

**Fig 1:**
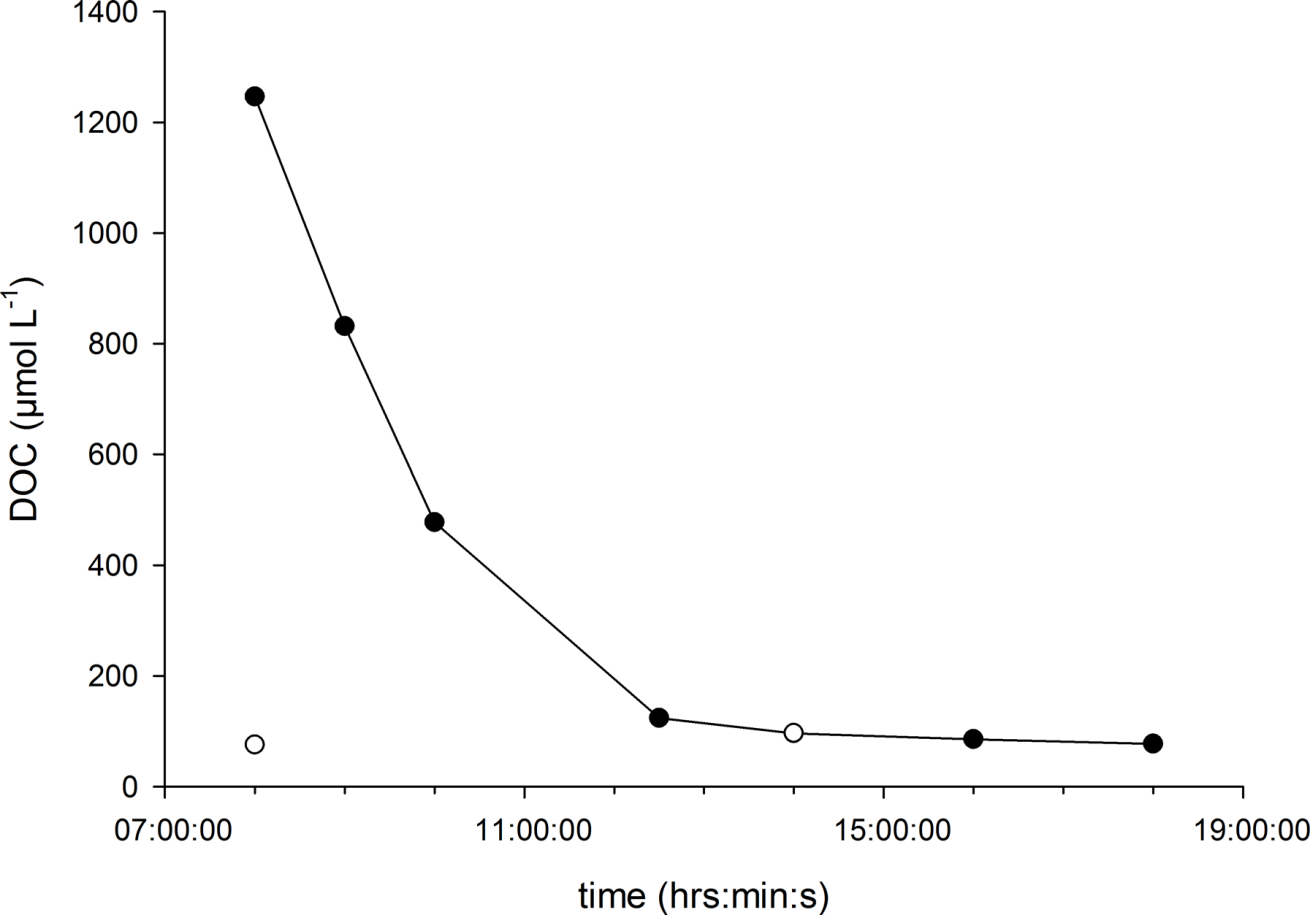
DOC concentrations in the high DOC treatment. Time series (08:00 am until 07:00 pm) after addition of 1170 μmol L^-1^ DOC as glucose (filled circles) and a background concentration (unfilled circles) of 76 and 97 μmol L^-1^ DOC. Filled circles indicate sampling points (n = 2) for DOC analysis of the high DOC treatment and unfilled circles of the controls (n = 2 for each point).

The high DOC treatment was achieved by daily additions of 1170 μmol L^-1^ DOC at 8:00 and 20:00 with pre- weighed Glucose (D-Glucose, Sigma Aldrich), a highly bioavailable short organic carbon molecule. This simulates a sudden increase of DOC and subsequent dissolution as likely to occur in coastal waters, along with sudden increases in river discharges that have been shown to correlate with high amounts of DOC [57–59]. Using stable DOC concentrations would not have adequately reflected natural conditions as production and recycling, especially of high bioavailable DOC occurs on a diurnal basis and even seasonal basis [60]. Dilution by flow through yielded an average concentration of 294 ± 506 μmol L^-1^ in the high DOC treatments determined over the seven sample points (n = 2) while background DOC concentrations of the low DOC treatments remained 83 ± 10 μmol L^-1^ (Fig 1).

The target *p*CO_2_ was 1000 μatm, corresponding to levels reached under the Representative Concentration Pathways (RCP) 6.0 to RCP 8.5 most likely by the year 2100 [61–63]. Calculated *p*CO_2_ levels yielded an average of 440 μatm *p*CO_2_ for control and 1090 μatm *p*CO_2_ for high DIC treatments (Table 1). DOC levels were chosen according to maximum concentrations measured in coral reefs (for summary see [32]) and treatments applied in other studies [32,33,54].

**Table 1:** Carbonate system parameters.

Treatment	pH	temp	Salinity	% O_2_Sat	TA	*p*CO_2_	HCO_3_^-^	ΩAr
	[Total]	[°C]	[ppt]		(μmol kgSW^-1^)	(μatm)	(μmol kgSW^-1^)	
**Control**	8.04	25.4	34.4	105.4	2276.1	402.8	1776.1	4.1
	(0.03)	(0.2)	(0.1)	(4.7)	(13.0)	(10.7)	(15.0)	(1.2)
**High DIC**	7.71	25.3	34.4	105.9	2281.2	995.9	2011.4	2.2
	(0.04)	(0.1)	(0.1)	(5.7)	(7.3)	(26.2)	(7.6)	(0.6)
**High DOC**	8.02	25.16	34.4	104.8	2286.4	429,8	1791.6	4.0
	(0.03)	(0.3)	(0.1)	(6.3)	(10.3)	(9.9)	(8.3)	(1.1)
**High DIC &**	7.69	25.0	34.4	104.2	2281.7	1080.5	2031.7	2.0
**High DOC**	(0.03)	(0.4)	(0.1)	(5.4)	(16.9)	(15.3)	(12.2)	(0.6)

Values calculated using CO2Calc with total alkalinity (TA) and pH_Total_ as input parameters (n = 3 for TA, n = 10 for pH, temperature, salinity and O_2_). Values are given as average with standard deviation in parentheses.

### Aquaria setup

The corals were kept in freshly filtered (0.5 μm) natural seawater at 25°C, with a salinity of 34.5. Water flow was adjusted to 150 mL min^-1^. Light was provided by individually adjustable white and blue light LED (6000 K, Aqua Illumination). Light levels were set equal to acclimation phase. Aquaria pumps (AquaWorld, Australia, 250 L h^-1^) in each specimen tank provided water movement. Target pH levels were achieved by a pH stat system (Aqua Medic, Germany) controlled by potentiometric pH sensors, as described in Vogel and Uthicke [64]. Total alkalinity A_T_ was determined by gran titration with a Metrohm 855 robotic titrosampler (Metrohm, Switzerland) using 0.5 M HCl (see Uthicke and Fabricius [18]) and certified reference material (CRM Batch 106, A. Dickson, Scripps Oceanographic Institute) for correction. Carbonate system parameters were calculated with CO2calc software [65] utilizing A_T_ values and pH values (Table 1) obtained with a multiprobe (WTW 3430, Germany).

### Maximum quantum efficiency

Maximum quantum efficiency (F_v_/F_m_) was determined by Pulse Amplitude Modulation (PAM) fluorometry using a diving PAM (Walz, Germany) and a 6 mm diameter fiber optic cable. F_v_/F_m_ measurements were conducted by light saturation pulse under steady fluoresces signals every evening, after dark adaptation, 30 min after the lights turned off automatically.

### Coral surface area

The individual surface area of the incubated coral nubbins from 5 to 16 cm^2^ was determined using the advanced geometry method [66]. Surface areas were calculated as individual columns, therefore height and width were measured using Image J software.

### Light-/ dark calcification, O_2_ and nutrient fluxes

After 16 d under experimental conditions, two coral nubbins from each replicate tank were transferred to individual closed plastic chambers (Nalgene 200 mL) and incubated for 60 min in light and 60 min in darkness. PH and DOC concentrations of the seawater in the incubation chambers corresponded to the formerly experienced treatment conditions, and the sea-water was pre-filtered some minutes before the start of each incubation in order to remove bacterial background signals from the incubation water. Each incubation run consisted of 12 parallel incubations in 200 mL closed chambers, including two blanks per treatment. A white light LED (4000 K, Megaman) was installed above each incubation chamber and individually adjusted to meet equal light conditions of the treatment tanks, verified using a quantum sensor (Apogee). To assure constant water temperature during incubation, chambers were placed into a temperature controlled water bath at 25°C, equal to the temperature during the 16 d incubation. Additionally, glass-coated magnetic stirrer bars ensured water movement within the incubation chambers.

Light- and dark calcification rates were determined by the alkalinity anomaly technique [67]. A subsample of 50 mL was pipetted from the incubation seawater and directly titrated for total alkalinity measurement by a Metrohm855 (as described above). A_T_ was calculated by non-linear regression fitting between pH 3.5 and pH 3.0. Calcium carbonate precipitation or dissolution in μM C h^-1^ was calculated by half molar of the difference between the post incubation and the blank seawater A_T_ readings, volume of chamber, time of incubation and organism surface area [68].

O_2_ production in light and consumption in darkness were monitored continuously during the incubations by three 4-channel O_2_ meters (Firesting, Pyroscience), connected to each chamber with fiber optic cables. Net photosynthesis, respiration, and resulting gross photosynthesis were determined in μM O_2_ h^-1^ and related to organism surface area. In addition, O_2_ consumption was corrected to blank readings from empty incubation chambers containing only the respective treatment water.

Nutrient fluxes in the chambers were determined by analyzing subsamples of seawater from light and dark incubations for dissolved inorganic nutrients, DIN (NH_4_^+^, PO_4_^-^ and NO_2_^-^ + NO_3_^-^ as NO_x_) and total organic carbon, TOC (NPOC) directly subsequent to the experimental runs. Samples for DIN were filtered using 0.45 μm syringe filters and kept frozen at -20°C until measurement by Segmented Flow Analysis (Seal Analytical). Samples for TOC were filtered through 0.45 μm GFF Filters (Whatman), acidified with 150 μL fuming HCl and frozen at -20°C until analysis on a Shimadzu TOC-5000A (Shimadzu). Nutrient fluxes in μM (DIN) and mg L^-1^ (TOC) were calculated and corrected for the fluxes of the blank incubations and related to organism surface area.

### Growth rates

Coral growth was determined using the buoyant weight technique [69]. Individual specimens were weighed (accuracy: 0.1 mg, Mettler Toledo) in a custom-build buoyant weight set-up with water jacket and seawater of constant temperature (25°C) and salinity (34.5) at the start and end of the experiment. All individuals of all treatments were measured the same day, therefore there was no need of using a standard. Growth of the organisms was expressed as daily percentage of weight change.

### Biological Oxygen Demand (BOD)

To assess effects of elevated organic or inorganic carbon availability on microbial respiration rates, BOD of the treatment water was measured at the end of the experiment for each treatment tank (n = 3). For this purpose, 200 mL of unfiltered seawater were incubated for 24 h in the dark under temperature conditions of the treatments. The O_2_ concentration (mg L^-1^ and % saturation) as well as salinity and temperature were recorded before and after the incubation, and O_2_ consumption rates were calculated from these two values and related to water volume and time to mg O_2_ L^-1^ h^-1^.

### Pigment content

Chl *a* content of *A*. *millepora* tissue was determined spectrophotometrically. After completion of the incubation experiments, organisms were frozen at -80°C. In the following, the protocol for Chl *a* measurement described in Vogel and Uthicke [64] and Schmidt et al. [70] was used. Coral tissue was separated from the skeleton by stripping with an air gun using fresh, ultra-filtered (0.2 μm) seawater. During several subsequent separation steps, the obtained zooxanthellae pellets were kept on ice for further processing, and the host tissue was frozen at -20°C for analysis of total protein content (as described below). Pellets were re-suspended in 5 mL of fresh, filtered seawater, and subsamples of 0.5 mL transferred into 2 mL centrifuge tubes. After centrifuging (10.000 x g for 5 min), the supernatant was discarded, and the zooxanthellae pellets were re-suspended in 2 mL of 95% EtOH to extract Chl *a*. Absorbencies were read in 400 μL of the supernatant on a 96-well microtiter plate at 750 and 665 nm wavelengths in a Powerwave microplate reader (BioTek). Chl *a* contents were calculated with equations by Nush [71] and related to nubbin surface area.

### Protein content

Total protein content of *A*. *millepora* was analyzed with the Bio-Rad protein assay kit (Bio-Rad). Applying the method described in Leuzinger et al. [72], the coral tissue slurry was digested with 1MNaOH for 60 min at 90°C in a sealed deep-well plate. Cell-debris was separated from the solution (1500 x g for 10 min). Dilutions of protein standard (bovine serum albumin, BSA) and samples were transferred into a 96-well microtiter plate and protein assay reagents were added. After 15 min, absorbency was read on 750 nm wavelength in a Powerwave microplate reader (BioTek). Total protein content of *A*. *millepora* was calculated, correlated to protein standard regression and related to nubbin surface area.

### Statistical analysis

We tested whether growth rates, light- and dark-calcification rates, photosynthesis, respiration, maximum quantum efficiency, pigment, protein content and nutrient fluxes differed significantly between treatments and control conditions. Data was tested for normality using the Shapiro-Wilk test and for equal variance using the Levene median test. Data of net and gross photosynthesis failed the test for equal variance, but showed equal variances after log10 transformation. A Two Way ANOVA was then performed with the treatments DIC and DOC as fixed factors to test for treatment effects as well as interactions of treatments and “aquarium” as nested factor to test for tank effects. To compare differences between individual treatment combinations, a Pairwise Multiple Comparison Procedure (Holm-Sidak method) was performed when interactions were significant. All statistical analyses were conducted using SigmaPlot 12.0 and NCSS statistical statistical software.

## Results

### Effects of DIC availability

**Fig 2:**
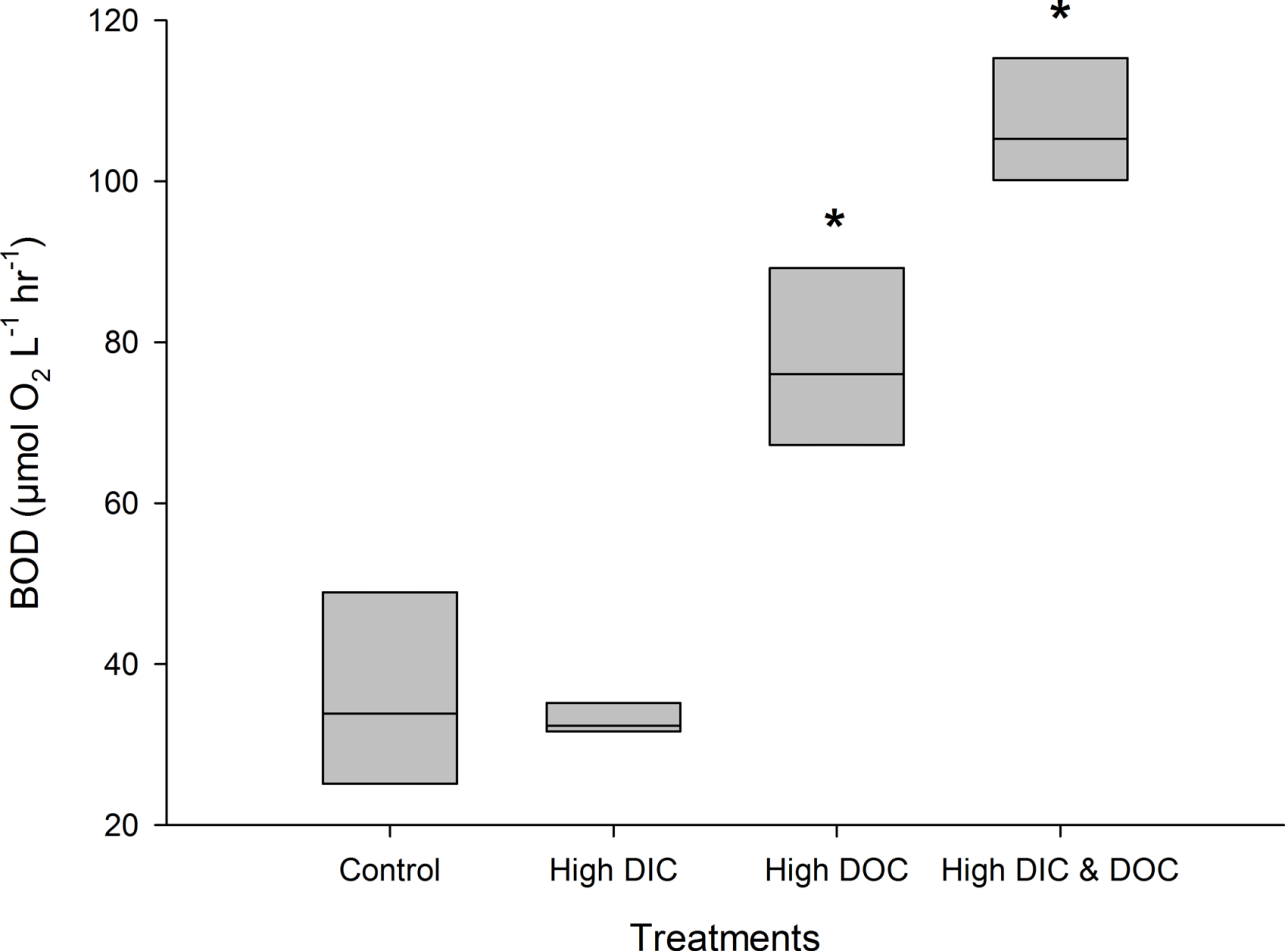
Biological Oxygen Demand (BOD) in the different treatments at the end of the experiment (n = 3). Data are compared between the control, the high DIC treatment (*p*CO_2_ 403 μatm), the high DOC treatment (DOC (added as Glucose 0 and 294 μmol L^-1^) and the combination of both treatments. Boxplots indicating median (mid of boxplot), 25% and 75% percentile (lower and upper border of boxplot). Significant differences (p < 0.05) are marked with an asterisk

**Fig 3:**
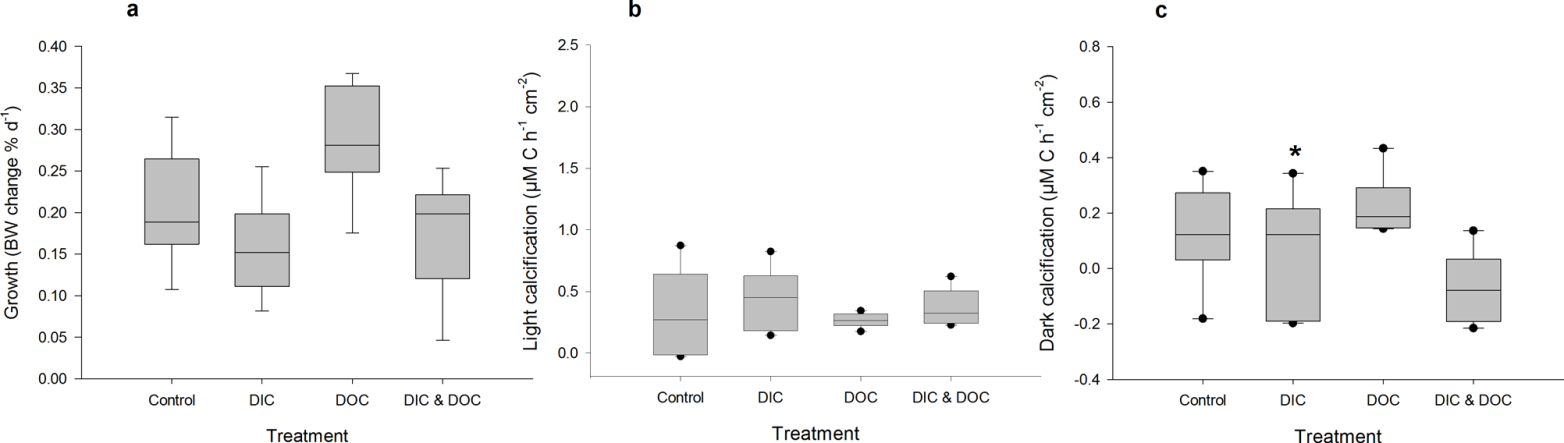
Physiological coral responses to treatments. Growth as (a) % change of buoyant weight (BW) of *Acropora millepora* (Ehrenberg, 1834) (n = 12). Calcification during light (b) (150 μmol photons*m^-2^s^-1^) and dark condition (c) measured via alkalinity anomaly technique and related to surface area. Data are compared between the control, the high DIC treatment (*p*CO_2_ 403 μatm), the high DOC treatment (DOC (added as Glucose 0 and 294 μmol L^-1^) and the combination of both treatments. Boxplots indicating median (mid of boxplot), 25% and 75% percentile (lower and upper border of boxplot) and 90 and 10% percentile (whiskers). Significant differences (p < 0.05) relative to controls are marked with an asterisk

**Fig 4:**
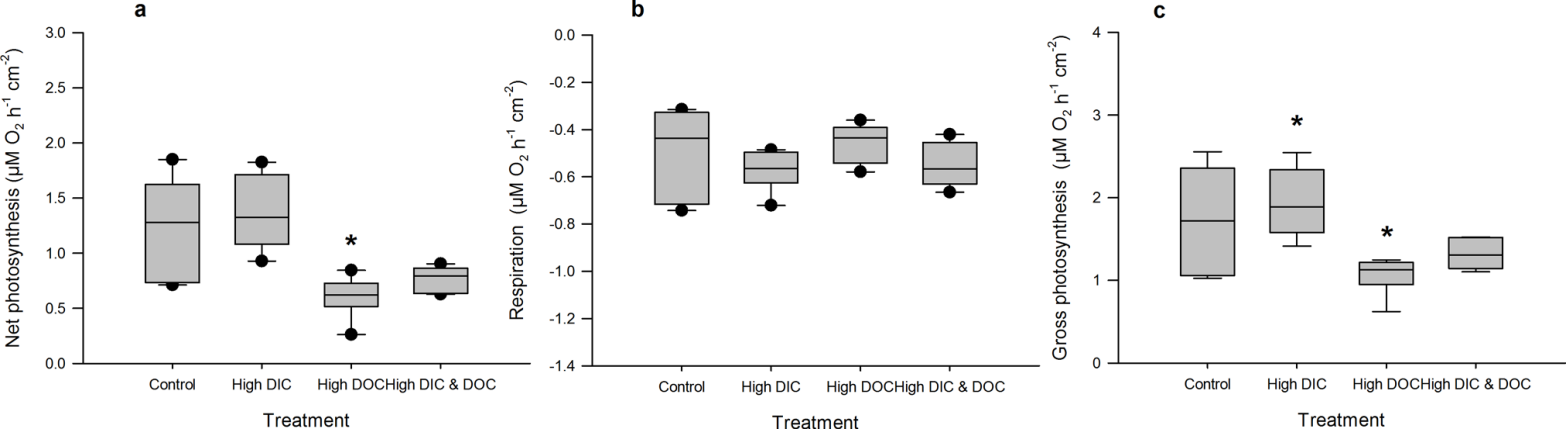
Oxygen fluxes as responses to treatments. Net photosynthesis (a), respiration (b) and gross photosynthesis (c) of *Acropora millepora* (Ehrenberg, 1834) (n = 6). Net photosynthesis measured during light (150 μ photons *m^-2^s^-1^) conditions and respiration during dark condition and related to coral surface area. Data are compared between the control, the high DIC treatment (*p*CO_2_ 403 μatm), the high DOC treatment (DOC (added as Glucose 0 and 294 μmol L^-1^) and the combination of both treatments. Boxplots indicating median (mid of boxplot), 25% and 75% percentile (lower and upper border of boxplot) and 90 and 10% percentile (whiskers). Significant differences (p < 0.05) are marked with an asterisk

**Fig 5:**
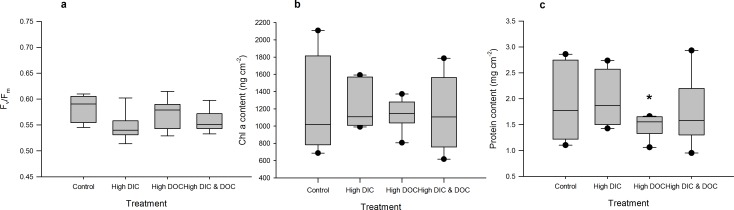
Photosystem parameters as response to treatments. Maximum quantum yield (a) of dark adapted individuals of *Acropora millepora* (Ehrenberg, 1834) (n = 12). Chlorophyll content (b) related to fresh weight (n = 6). Protein content (c) of *A*. *millepora* related to surface area (n = 6). Data are compared between the control, the high DIC treatment (*p*CO_2_ 403 μatm), the high DOC treatment (DOC (added as Glucose 0 and 294 μmol L^-1^) and the combination of both treatments. Boxplots indicating median (mid of boxplot), 25% and 75% percentile (lower and upper border of boxplot) and 90 and 10% percentile (whiskers). Significant differences (p < 0.05) are marked with an asterisk

**Fig 6:**
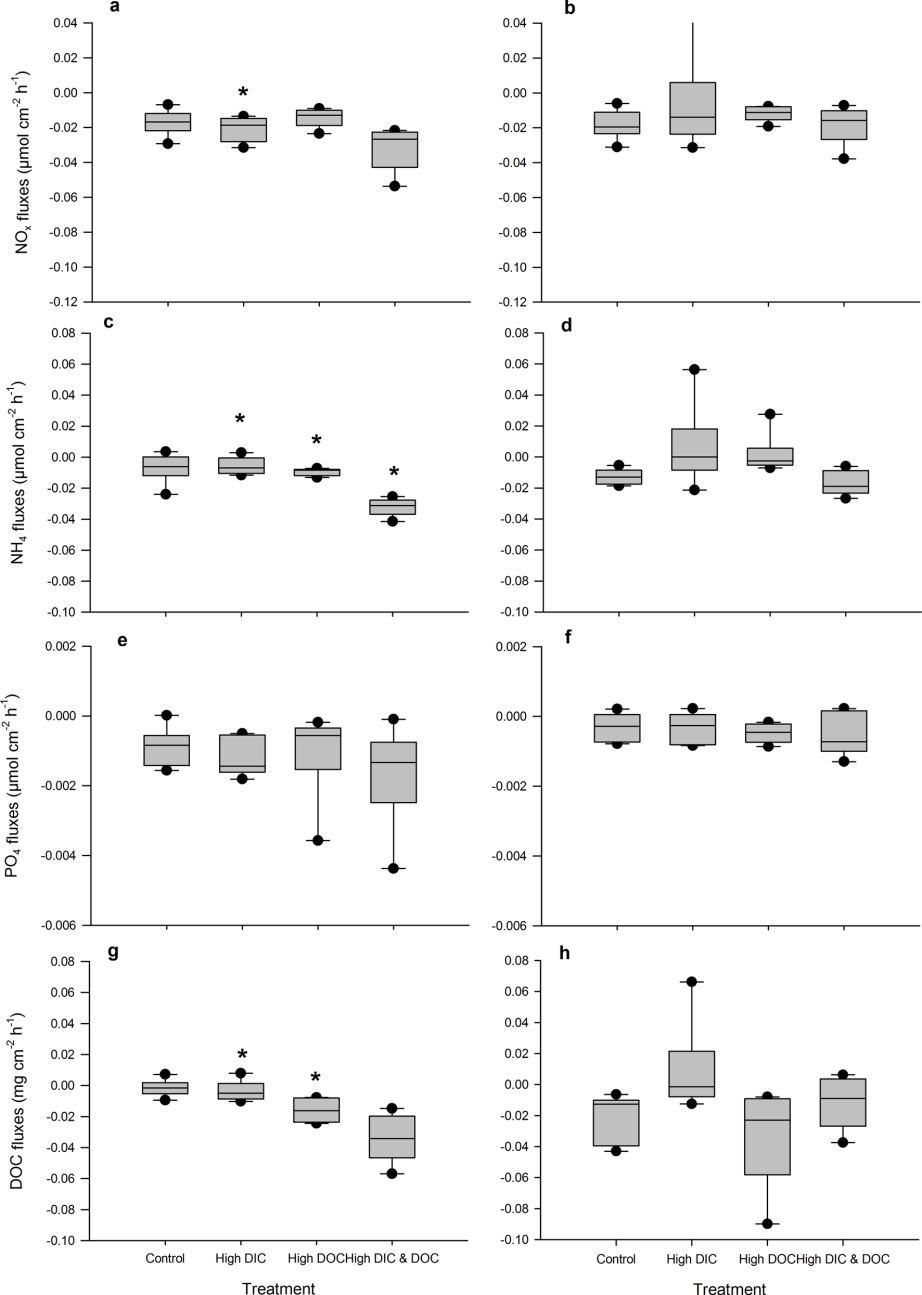
Nutrient fluxes as response to treatments. NO_x_ fluxes (a, b), NH_4_^+^ fluxes (c, d) PO_4_^-^ fluxes (e, f) and DOC fluxes (g, h) calculated from light (150 μmol photons *m^-2^s^-1^) (graphs on the left) and dark incubations (graphs on the right) of *Acropora millepora* (Ehrenberg, 1834) (n = 12). Data are compared between the control, the high DIC treatment (*p*CO_2_ 403 μatm), the high DOC treatment (DOC (added as Glucose 0 and 294 μmol L^-1^) and the combination of both treatments. Boxplots indicating median (mid of boxplot), 25% and 75% percentile (lower and upper border of boxplot) and 90 and 10% percentile (whiskers). Significant differences (p < 0.05) are marked with an asterisk

High DIC availability did not affect the BOD of the treatment water compared to controls (Fig 2). It significantly reduced dark calcification rates of *A*. *millepora* by 50% with 0.06 μmol C cm^-2^ h^-1^ compared to 0.13 μmol C cm^-2^ h^-1^ in the control treatments (Fig 3C, S1 Table), but did not affect calcification in light (Fig 3B). High DIC also reduced growth of *A*. *millepora* by 23% with 0.2% bw d^-1^ compared to 0.16% bw d^-1^ in the controls (Fig 3A). In contrast, respiration rates as well as net and gross photosynthesis were not affected by high DIC (Fig 4A–4C), although photochemical efficiency was significantly reduced by 6% from 0.58 to 0.55 (Fig 5A). Chl *a* and protein (Fig 5B and 5C) contents of *A*. *millepora* were not affected by high DIC availability. High DIC availability did however significantly increase NO_x_ uptake of the coral under light conditions by 21% from 0.017 to 0.021 μmol cm^-2^ h^-1^ (Fig 6A), while this was not the case under dark conditions (Fig 6B). Neither NH_4_^+^ nor PO_4_^-^ uptake was influenced by high DIC availability under light or dark conditions (Fig 6C–6F). In contrast, DOC release was stimulated through high DIC availability by 141% from 1.75 to 0.75 μmol cm^-2^ h^-1^, but only in dark conditions (Fig 6G and 6H).

### Effects of DOC availability

High DOC availability significantly increased BOD by 115% from 0.81 to 1.73 mg L^-1^ h^-1^ (Fig 2). It did not affect the calcification rate measured during light or dark incubation (Fig 3B and 3C), but increased growth by 42% from 0.20 to 0.29% bw d^-1^ compared to the controls (Fig 3A). The high DOC treatment also reduced net photosynthesis by 51% from 1.24 to 0.63 μmol O_2_ cm^-2^ h^-1^ (Fig 4B) and gross photosynthesis by 39% from 1.73 to 1.06 μmol O_2_ cm^-2^ h^-1^(Fig 4C). High DOC availability did not affect respiration rates of *A*. *millepora* (Fig 4A). Chl *a* and protein content along with photosynthetic efficiency were unaffected (Fig 5A–5C). The same was observed for NO_x_ fluxes (Fig 6A and 6B). Ammonium uptake however was increased by 36% from 0.007 to 0.009 μmol cm^-2^ h^-1^ (Fig 6C) during light conditions, but not during dark conditions. The latter was also true for PO_4_^-^ fluxes (Fig 6D–6F). DOC uptake rates were only affected in light incubations and increased by 927% from 0.17 to 1.3 μmol cm^-2^ h^-1^ (Fig 6G and 6H).

### Combined effects of DIC and DOC availability

The combined high DIC and high DOC treatments led to additive and interactive effects on some of the variables measured. BOD increased further by another 81% compared to the DOC treatment and 197% compared to the controls, with 2.39 mg L^-1^ h^-1^ (Fig 2) indicating a synergistic effect.

Coral growth under combined high DOC and DIC availability was similar compared to control conditions, but significantly lower than under high DOC conditions alone and not significantly higher than under high DIC treatment (Fig 3A). No significant change was observed for light calcification under the combined treatment (Fig 3B). In contrast, dark calcification was reduced by 105% compared to the DIC treatment and 150% compared to the control conditions (Fig 3C). High DOC and DIC did not show any combined effects on photosynthesis and respiration (Fig 4A–4C). The same was observed for photosynthetic efficiency, Chl *a* content and protein content (Fig 5A–5C). The uptake rates of NO_x_ in the combined treatment compared to the controls and the DIC treatment were increased in light by 65% and 86%, respectively (Fig 6A). In the dark however, no significant differences were observed (Fig 6B). For NH_4_^+^ uptake rates, similar results were observed in both dark and light incubations (Fig 6C and 6D). In light, NH_4_^+^ uptake rates increased by 330% compared to the DOC treatment and 366% compared to the control. In the dark, NH_4_^+^ uptake rates increased by 75% compared to the DIC treatment and 100% compared to the controls. No such trend was observed for the uptake rates of PO_4_^-^ (Fig 6E and 6F). However, in light the uptake rates of DOC in the combined treatment were increased by 1163% compared to the DOC treatment and by 2090% compared to the controls (Fig 6G). In the dark incubations, DOC uptake rates were not affected by high DOC and DIC concentrations (Fig 6H).

## Discussion

### The effects of high DIC availability

The BOD in the incubation chambers, an indicator of bacterial respiration during the experiment, did not change under high DIC conditions (Table 2). This is consistent with a study on microbial biofilms from the GBR that also reported constant bacterial background respiration and nutrient fluxes, despite different DIC levels [73]. Hence, bacterial communities either did not change during exposure to elevated DIC or rapidly acclimated as suggested by Witt et al. [73]. High DIC did not affect light calcification and photosynthesis of *A*. *millepora*, but decreased dark calcification and growth. This contrasts with a previous study on an acroporid coral that reported a reduction of photosynthetic productivity under elevated DIC conditions [14]. However, in the before mentioned study, high light dosages of up to 1200 μmol photons m^-2^ s^-1^ were used that induced bleaching and consecutively productivity loss. In the present experiment, the continuous light dose of 150 μmol photons m^-2^ s^-1^ was comparable to studies under natural light regimes that found no effect of elevated DIC on the productivity of corals [74,75]. We observed carbonate dissolution under dark incubations (Table 2), which corroborates previous studies that demonstrated negative effects of elevated DIC on coral growth and a net dissolution under similar high *p*CO_2_ levels [9,14,28]. The *p*CO_2_ levels of the high DIC treatment corresponded to set treatment conditions as aimed for [63], and control conditions were close to present-day levels [76] or below levels observed in inshore reefs with higher natural variability [30], but in *A*. *millepora* dissolution occurred mainly under dark conditions.

**Table 2:** Summary of main effects.

Treatment	Photosynthesis		Respiration	Calcification		Growth
	net	gross		light	dark	
**High DIC**	No effect	No effect	No effect	No effect	Reduced (50%)	Reduced (23%)
**High DOC**	Reduced (51%)	Reduced (39%)	No effect	No effect	No effect	Reduced (42%)
**High DIC &**	No effect	No effect	No effect	No effect	Reduced (50%)	No effect
**High DOC**						

Effects as revealed by mixed model ANOVA for photosynthesis, respiration, calcification, and growth are summarized by treatments. Relative values (%) compared to control conditions are given for significant effects.

We show that the effect of ocean acidification on calcification becomes most visible when no photosynthetic activity was present in dark conditions. This supports recent findings that show that respiratory processes may enhance the negative effects of elevated DIC concentrations and are the main cause of reduced growth [31]. We found no change in Chl *a* and protein content which explains the stable productivity under elevated DIC concentrations. However, we recorded a significant reduction in photochemical efficiency, eventually also leading to reduced photosynthetic rates. During the 16 d experiment, we could detect the decline of photochemical efficiency, but no acclimation. Long-term experiments could reveal the cause of a reduced productivity and evaluate potential acclimation.

To our knowledge, the present study is the first to reveal that high DIC stimulates the uptake of NO_x_ and the release of DOC of corals. The uptake rate of NO_x_ under control conditions lies within described uptake rates for the genus *Acropora*, although nitrate concentrations were slightly elevated (1.3 μM) compared to other studies (e.g. Bythell 1990, 0.22–1.72 μM). The induced NO_x_ uptake probably resulted from a higher demand for nitrogen to allow keeping productivity stable and on a high level, despite reduced photochemical efficiency. The observed increased DOC release on the other hand may have been caused by high availability of bicarbonate for photosynthesis ensuing increased carbon release when nitrate uptake rates were lower and nitrate becomes limiting [77].

### The effects of high DOC availability

In contrast to the high DIC treatment, BOD values in the incubation chambers increased under high DOC. This is in line with other experimental studies investigating the responses of micro-organisms to elevated DOC [32,33,46]. While high DOC reduced photosynthesis of *A*. *millepora*, it did not affect coral light or dark calcification, but significantly increased coral net growth (Table 2). The reduced photosynthesis rates are likely caused by the high microbial respiration of the coral host with reduced pH of the water directly on the coral surface compared to the surrounding water. The observed increased net growth of the coral under high DOC availability probably originated from heterotrophic compensation of losses in assimilates due to reduced photosynthesis and the surplus of bio-available organic carbon as energy source. This is supported by the finding that bleached corals can survive through increased heterotrophic feeding [78,79] and are able to maintain photosynthetic quantum yield during thermal stress [80]. Bleached corals can restore dark calcification when glycerol is added [81], and unbleached corals showed increased calcification rates under glucose addition [82].

The utilization of both ammonia and DOC during light hours under elevated DOC availability may indicate increased microbial activity via ammonia oxidation and carbohydrate metabolism, rather than direct uptake by the coral host itself as supported by the significantly increased BOD. In contrast to other studies, no signs of disease or bleaching occurred during the present study. Although the levels of DOC applied were increased by 500% on average, they were low compared to other studies [32,33,54]. The pH under the high DOC concentration was low, indicating higher bacterial activity as confirmed by a reduction in oxygen saturation, the increase in bicarbonate ions and consequently a reduction of the aragonite saturation state.

The addition of glucose twice a day was chosen to mimic natural fluctuation of bioavailable DOC as occurring during periods of heavy rainfall and river input which correlates to high DOC concentrations [57–59]. Furthermore, availability of bioavailable DOC does not only change within season and time of the day [60], but DOC is also readily taken up by e.g. corals themselves and mainly bacteria [52,60,83]. The total level of glucose added is comparable to other studies, where monosaccharides were added [32,33,54] and was on the higher end of DOC concentrations described for different reef settings in high DOC environments [32]. In the present experimental study, we used glucose to increase DOC to assure reproducibility. Future studies should now evaluate the effects of combined sugars or different algal exudates in combination with ocean acidification. The latter approach however would need to consider that concentrations of algae- derived DOC change under different light conditions and are dependent on the species of algae [53,60].

### The combined effects of high DIC and DOC availability

The combination of both high DIC and DOC availability in this study led to a higher BOD compared to the DOC treatment effect alone (Table 2). This synergistic effect [84] was probably caused by altered, heterotrophic bacterial communities due to a higher stress reaction of the coral towards high DIC and DOC, as described for elevated DIC [85] or DOC availability alone [32]. The combination of both factors significantly decreased dark calcification, increased ammonia, NO_x_ and DOC uptake rates, but did not affect photosynthesis, light calcification or growth. The present study revealed that high DIC and DOC availability has additive negative effects, and the dark calcification was further reduced under the combined treatment than at high DIC levels alone. This may be due to the elevated bacterial respiration under high DOC conditions, which increased the DIC concentration locally above the level of the DIC treatment condition. Hence, the negative impact of the DIC treatment alone was potentially further enhanced due to respiratory processes as mentioned by other studies on DIC effects alone [31]. Overall, growth was similar to the control conditions and likely affected by the surplus of energy for calcification from the DOC treatment, thereby balancing the DIC treatment effect.

The increase in uptake rates of ammonia, NO_x_ and DOC under the combined treatment compared to the individual treatments and the control was likely caused by higher bacterial activity, ammonia oxidation, and nitrate uptake. However, it may also be a sign of shifts in microbial community structure due to excess energy derived from high DOC availability. Additionally, the combined effects of both treatments on the coral host may disrupt ‘natural’ bacterial-host interactions. Further studies should now include longer experimental periods as well as stepwise or gradual increase of exposure to both treatments.

### Ecological perspective

Our experiment is the first to show that high DOC availability positively impacted coral growth. On the other hand, this study reveals that high DIC negatively affects coral carbonate production because of dissolution at night. The high DIC/DOC combination treatment amplified this negative effect, leading to further decreased carbonate production. Thus, the results of the present study strongly suggest that the simultaneous occurrence of high DIC and DOC in present coastal waters, and likewise even more in future waters, constitutes a serious threat to corals. This will likely negatively influence their ecological functions and services, e.g. habitat provisioning and coastal protection.

The present study further demonstrates that BOD increases under high DOC availability, and even stronger increases under combined high DIC/DOC concentrations. DOC concentrations lower than used in this study can strongly increase bacterial oxygen consumption in the water, and higher concentrations may even cause local oxygen deficiency and coral death [32,86]. In the context of global change and increasing land-derived pollution, DOC concentrations and BOD are strong first indicators for shifts in bacterial regimes. This study thus suggests considering both parameters when assessing reef health in monitoring programs. The control and reduction of labile, highly bio-available DOC from land-derived sources through water management needs to be taken into account when mitigating potential effects of riverine inputs on coral reefs.

## Supporting Information

S1 Table Results of Two Way ANOVA for DIC and DOC as fixed factors and “aquarium” as nested factor. Degree of freedom (DF), sum of squares (SS) and mean square (MS) as well as F and P values are given. Data for gross and net photosynthesis were log10 transformed prior to analysis. Significant results are marked bold with an asterisk (*).
(DOCX)
