# Supplementary material for: Effects of High Dissolved Inorganic and Organic Carbon Availability on the Physiology of the Hard Coral Acropora millepora from the Great Barrier Reef
Source: PLoS One. 2016 Mar 9;11(3):e0149598. doi: 10.1371/journal.pone.0149598 (PMC4784739; doi:10.1371/journal.pone.0149598)
Supplement: S1 Table — Degree of freedom (DF), sum of squares (SS) and mean square (MS) as well as F and P values are given. Data for gross and net photosynthesis were log10 transformed prior to analysis. Significant results are marked bold with an asterisk (*). (DOCX) [file pone.0149598.s001.docx]

**S1 Table.** **Results of Two Way ANOVA for DIC and DOC as fixed factors and “aquarium” as nested factor.**

| **Response variable** | **Source of variation** | **DF** | **SS** | **MS** | **F-value** | **P-value** |
| --- | --- | --- | --- | --- | --- | --- |
| **Biological oxygen** | DIC | 1 | 0.265 | 0.265 | 6.377 | **0.036*** |
| **demand** | DOC | 1 | 5.009 | 5.009 | 120.766 | **<0.001*** |
|  | DIC x DOC | 1 | 0.394 | 0.394 | 9.510 | 0.015 |
|  | Residual | 9 | 0.332 | 0.0415 |  |  |
|  | Total | 11 | 6.000 | 0.545 |  |  |
| **Growth** | DIC | 1 | 0.0676 | 0.0676 | 4.8 | 0.0598 |
|  | DOC | 1 | 0.0289 | 0.0289 | 5.07 | 0.054 |
|  | DIC x DOC | 1 | 0.0114 | 0.0114 | 0 | 0.984 |
|  | Aquarium | 8 | 0.0760148 | 0.009501854 | 2.9 | **0.015*** |
|  | Residual | 31 | 0.157 | 0.00412 |  |  |
|  | Total | 41 | 0.267 | 0.00651 |  |  |
| **Light calcification** | DIC | 1 | 0.0631 | 0.0631 | 0.90 | 0.369 |
|  | DOC | 1 | 0.0243 | 0.0243 | 0.35 | 0.571 |
|  | DIC x DOC | 1 | 0.000472 | 0.000472 | 0.01 | 0.936 |
|  | Aquarium | 8 | 0.5581167 | 0.06976458 | 1.65 | 0.218 |
|  | Residual | 11 | 1.024 | 0.0539 |  |  |
|  | Total | 22 | 1.116 | 0.0507 |  |  |
| **Dark calcification** | DIC | 1 | 0.191 | 0.191 | 8.08 | **0.0217*** |
|  | DOC | 1 | 0.00201 | 0.00201 | 0.08 | 0.778 |
|  | DIC x DOC | 1 | 0.0787 | 0.0787 | 3.32 | 0.106 |
|  | Aquarium | 8 | 0.1894387 | 0.023679 | 0.83 | 0.593 |
|  | Residual | 12 | 0.532 | 0.0266 |  |  |
|  | Total | 23 | 0.804 | 0.0350 |  |  |
| **Net** | DIC | 1 | 0.0496 | 0.0496 | 2.567 | 0.125 |
| **photosynthesis** | DOC | 1 | 0.453 | 0.453 | 23.445 | **<0.001*** |
|  | DIC x DOC | 1 | 0.00572 | 0.00572 | 0.296 | 0.592 |
|  | Aquarium | 8 | 0.1269225 | 0.0158653 | 0.73 | 0.662 |
|  | Residual | 12 | 0.387 | 0.0193 |  |  |
|  | Total | 23 | 0.895 | 0.0389 |  |  |
| **Respiration** | DIC | 1 | 0.0439 | 0.0439 | 2.957 | 0.101 |
|  | DOC | 1 | 0.00527 | 0.00527 | 0.355 | 0.558 |
|  | DIC x DOC | 1 | 0.000457 | 0.000457 | 0.0308 | 0.736 |
|  | Aquarium | 8 | 0.07187044 | 0.008983805 | 0.48 | 0.849 |
|  | Residual | 12 | 0.297 | 0.0148 |  |  |
|  | Total | 23 | 0.346 | 0.0151 |  |  |
| **Gross** | DIC | 1 | 0.0419 | 0.0419 | 5.68 | **0.044*** |
| **photosynthesis** | DOC | 1 | 0.193 | 0.193 | 26.14 | **<0.001*** |
|  | DIC x DOC | 1 | 0.00158 | 0.00158 | 0.21 | 0.592 |
|  | Aquarium | 8 | 0.0590336 | 0.007379199 | 0.42 | 0.886 |
|  | Residual | 12 | 0.269 | 0.0134 |  |  |
|  | Total | 23 | 0.505 | 0.0220 |  |  |
| **Maximum quantum yield** | DIC | 1 | 0.00699 | 0.00699 | 4.67 | 0.0627 |
|  | DOC | 1 | 0.00000307 | 0.00000307 | 0.53 | 0.486 |
|  | DIC x DOC | 1 | 0.00146 | 0.00146 | 0 | 0.976 |
|  | Aquarium | 8 | 0.02665 | 0.003332 | 1.38 | 0.237 |
|  | Residual | 36 | 0.0286 | 0.000666 |  |  |
|  | Total | 47 | 0.0370 | 0.000804 |  |  |
| **Chlorophyll a content** | DIC | 1 | 287.783 | 287.783 | 0.00187 | 0.962 |
|  | DOC | 1 | 41418.681 | 41418.681 | 0.269 | 0.573 |
|  | DIC x DOC | 1 | 204.592 | 204.592 | 0.00133 | 0.968 |
|  | Aquarium | 8 | 961368.1 | 120171 | 0.68 | 0.700 |
|  | Residual | 12 | 2116668.558 | 176389 |  |  |
|  | Total | 23 | 3119946.614 | 135649.853 |  |  |
| **Protein content** | DIC | 1 | 0.164 | 0.164 | 1.32 | 0.283 |
|  | DOC | 1 | 0.699 | 0.699 | 5.63 | **0.045*** |
|  | DIC x DOC | 1 | 0.0460 | 0.0460 | 0.37 | 0.559 |
|  | Aquarium |  | 0.993662 | 0.1242077 | 0.26 | 0.968 |
|  | Residual | 20 | 6.775 | 0.339 |  |  |
|  | Total | 23 | 7.684 | 0.334 |  |  |
| **NO_X_ fluxes** | DIC | 1 | 0.000666 | 0.000666 | 21.93 | **0.006*** |
| **in light** | DOC | 1 | 0.000103 | 0.000103 | 3.41 | 0.243 |
|  | DIC x DOC | 1 | 0.000289 | 0.000289 | 9.46 | **0.0152*** |
|  | Aquarium | 8 | 0.000243229 | 0.0000304036 | 0.31 | 0.948 |
|  | Residual | 12 | 0.00143 | 0.0000716 |  |  |
|  | Total | 23 | 0.00249 | 0.000108 |  |  |
| **NO_X_ fluxes** | DIC | 1 | 0.0000276 | 0.0000276 | 5.2 | 0.051 |
| **in dark** | DOC | 1 | 0.0000328 | 0.0000328 | 6.24 | **0.037*** |
|  | DIC x DOC | 1 | 0.000456 | 0.000456 | 1.99 | 0.195 |
|  | Aquarium | 8 | 0.08068237 | 0.0100853 | 0.11 | 0.997 |
|  | Residual | 12 | 0.00430 | 0.000215 |  |  |
|  | Total | 23 | 0.00481 | 0.000209 |  |  |
| **NH_4_ fluxes** | DIC | 1 | 0.000697 | 0.000697 | 17.813 | **<0.001*** |
| **in light** | DOC | 1 | 0.00126 | 0.00126 | 32.288 | **<0.001*** |
|  | DIC x DOC | 1 | 0.000867 | 0.000867 | 22.162 | **<0.001*** |
|  | Aquarium | 8 | 0.0003170658 | 0.00003963 | 1.03 | 0.466 |
|  | Residual | 12 | 0.000782 | 0.0000391 |  |  |
|  | Total | 23 | 0.00361 | 0.000157 |  |  |
| **NH_4_ fluxes** | DIC | 1 | 0.0000000663 | 0.0000000663 | 0.000280 | 0.987 |
| **in dark** | DOC | 1 | 0.000117 | 0.000117 | 0.495 | 0.490 |
|  | DIC x DOC | 1 | 0.00210 | 0.00210 | 8.30 | **0.0205*** |
|  | Aquarium | 8 | 0.002020055 | 0.000252506 | 1.13 | 0.416 |
|  | Residual | 12 | 0.00474 | 0.000237 |  |  |
|  | Total | 23 | 0.00695 | 0.000302 |  |  |
| **PO_4_ Fluxes** | DIC | 1 | 0.00000139 | 0.00000139 | 1.278 | 0.272 |
| **in light** | DOC | 1 | 0.000000491 | 0.000000491 | 0.451 | 0.509 |
|  | DIC x DOC | 1 | 0.000000150 | 0.000000150 | 0.138 | 0.715 |
|  | Aquarium | 8 | 0.001739491 | 0.0002174363 | 1.06 | 0.446 |
|  | Residual | 12 | 0.0000218 | 0.0002048563 |  |  |
|  | Total | 23 | 0.0000238 | 0.00000104 |  |  |
| **PO_4_ Fluxes** | DIC | 1 | 0.00000964 | 0.00000964 | 0.898 | 0.355 |
| **in dark** | DOC | 1 | 0.0000136 | 0.0000136 | 1.270 | 0.273 |
|  | DIC x DOC | 1 | 0.0000107 | 0.0000107 | 0.75 | 0.330 |
|  | Aquarium | 8 | 2.993147 | 0.3741434 | 0.99 | 0.486 |
|  | Residual | 12 | 0.000215 | 0.0000107 |  |  |
|  | Total | 23 | 0.000249 | 0.0000108 |  |  |
| **DOC fluxes** | DIC | 1 | 0.000600 | 0.000600 | 16.23 | **0.003*** |
| **in light** | DOC | 1 | 0.00303 | 0.00303 | 81.93 | **<0.001*** |
|  | DIC x DOC | 1 | 0.000389 | 0.000389 | 10.55 | **0.011*** |
|  | Aquarium | 8 | 0.0002962058 | 0.00003702 | 0.29 | 0.956 |
|  | Residual | 12 | 0.00184 | 0.0000919 |  |  |
|  | Total | 23 | 0.00586 | 0.000255 |  |  |
| **DOC fluxes** | DIC | 1 | 0.00393 | 0.00393 | 4.63 | 0.063 |
| **in dark** | DOC | 1 | 0.00162 | 0.00162 | 1.91 | 0.203 |
|  | DIC x DOC | 1 | 0.0000808 | 0.0000808 | 0.09 | 0.767 |
|  | Aquarium | 8 | 0.006790499 | 0.0008488124 | 2.01 | 0.133 |
|  | Residual | 12 | 0.0119 | 0.000593 |  |  |
|  | Total | 23 | 0.0175 | 0.000761 |  |  |
